# AdBSK1-Mediated Hormone Signaling Regulates Flowering Transition in *Actinidia deliciosa* ‘*Guichang*’

**DOI:** 10.3390/genes16070760

**Published:** 2025-06-28

**Authors:** Lina Guo, Xiaoyu Cui, Jiayin Li, Chao Zhang, Yumei Fang

**Affiliations:** 1 School of Biological Science and Technology, Liupanshui Normal University, Liupanshui 553004, China; guolina@lpssy.edu.cn (L.G.); zhangchaoscu@126.com (C.Z.); 2 College of Life Science, Northwest A&F University, Xianyang 710100, China; cuixiaoyucxycxy@163.com (X.C.); l_ges1111@163.com (J.L.)

**Keywords:** kiwifruit, ‘*Guichang*’, bud, flowering, transcriptomics, proteomics

## Abstract

Background: The *Actinidia deliciosa cultivar* ‘*Guichang*’ is a remarkable kiwifruit variety. The phenotypic traits of this variety are influenced by the climatic conditions in Guizhou. The flowering time, which is shaped by multiple environmental factors, has a substantial impact on both the fruit yield and quality. Objectives and Methods: This study was designed to explore the molecular mechanisms underlying the transition from bud to flowering in ‘*Guichang*’ through transcriptomic and proteomic analyses. Results: The transcriptomic results revealed that 6201 genes were up-regulated, while 5849 genes were down-regulated during this transition. Key genes related to hormone signaling, such as *AdPIF4*, *AdBSK*, *AdBRI1*, and *AdCYCD3*, were recognized as crucial regulators. The proteomic analysis detected a total of 10,488 proteins. Among them, AdBSK1 was regulated, while AdPIF4, AdBRI1, and AdCYCD3 showed stable expressions. A moderate positive correlation (with a Pearson correlation coefficient of 0.445) was found between the expression levels of transcriptomics and proteomics. When AdBSK1 was over-expressed in Arabidopsis, it promoted earlier flowering. This was achieved by down-regulating *FLC* and up-regulating *FT* and *SOC1*. Conclusions: This study clarifies the molecular mechanisms involved in the bud-to-flowering transition in ‘*Guichang*’. It emphasizes the intricate interactions among hormonal pathways, key genes, and proteins, which are consistent with the broader understanding of plant flowering regulation in recent research. These findings are significant for deepening our understanding of, and potentially controlling, the flowering mechanisms.

## 1. Introduction

The *Actinidia deliciosa cultivar* ‘*Guichang*’, commonly known as ‘*Guichang*’ kiwifruit, is celebrated for its high quality and is often used in tissue culture systems to propagate virus-free plants [1]. The Guizhou region, with its unique climate marked by significant daily temperature changes, high ultraviolet radiation, and plentiful rainfall, enhances the fruit’s distinctive flavor and boosts the levels of folic acid (vitamin B9) and vitamin C. These conditions also ensure a consistent fruit size and vivid color, making it a great addition to a healthy diet [2]. However, variations in the flowering time of ‘*Guichang*’, affected by environmental factors, can influence the pollination efficiency and fruit setting, impacting the overall yield and quality [3,4,5,6].

The process of plants transitioning from bud development to flowering is intricate and demands the coordination of various molecular mechanisms. Prior research has indicated that numerous genes, signaling pathways, and environmental factors work together to regulate the timing and manner of this critical transition [7,8,9]. Many studies have shown that gibberellin (GA) and brassinosteroids (BRs) are essential for the regulation of flowering [10]. Meanwhile, PIF3/4 (Phytochrome Interacting Factor 3/4) is involved in temperature regulation. This regulation impacts the expression of FT and SOC1, thereby influencing flowering timing [11,12].

In the brassinosteroid biosynthesis pathway, BRI1 (Brassinosteroid Insensitive 1) functions as the receptor kinase and forms a heterodimer with BAK1 (BRI1-Associated Receptor Kinase 1), activating downstream signaling pathways. Activated BRI1 phosphorylate BSKs are important for plant growth and development [13]. BSK1 (Brassinosteroid-Signaling Kinase 1) and BSK2 (Brassinosteroid-Signaling Kinase 2) have been shown to positively regulate flower organ abscission, independently of BSK1’s kinase activity [14]. Phosphorylated BSKs activate BSU1 phosphatase while inhibiting BIN2 kinase. When BIN2 is inhibited, BZR1 (Brassinazole-Resistant 1) and BES1 (BRI1-EMS-Suppressor 1) are dephosphorylated, which promotes growth processes such as cell elongation [15,16,17]. BZR1, a key transcription factor in the BR signaling pathway, binds to the promoter of CONSTANS (CO). This binding suppresses the transcription of CO and indirectly enhances the expression of FT, ultimately accelerating the flowering process [18]. In *Arabidopsis*, BES1, a homology of BZR1, directly activates FT transcription, forming a regulatory module with BZR1-CO-FT that affects photoperiod-dependent flowering [19]. Furthermore, BZR1 interacts with light signaling factors such as PIF4, integrating BR signals with photoperiod and temperature signals [20,21]. BZR1 also collaborates with the blue light receptors CRY1/2 (CRYPTOCHROME 1/2) and the UV receptor UVR8 (UV RESISTANCE LOCUS 8), regulating parthenogenesis and flowering time [22,23]. In summary, the transition from bud to flower is a complex process influenced by various molecular mechanisms, with many specific pathways still needing further investigation.

Transcriptomics and proteomics are key omics technologies. Transcriptomics delves into the mechanisms of gene transcription, whereas proteomics provides deep insights into protein expression patterns and the intricacies of post-translational modifications. By combining these analyses, a detailed regulatory network of gene expression can be developed, highlighting the intricate interactions among regulatory elements like transcription factors, miRNAs, and their target genes, as well as their impact on the flowering processes of plants [24,25,26]. These essential genes and proteins are likely to be crucial in the initiation, development, or regulation of plant flowering, with further functional studies clarifying their specific contributions [27,28,29].

## 2. Methods and Materials

### 2.1. Plant Material Collection and Sample Preparation

Samples of buds and flowers were collected from 10-year-old kiwifruit plants in Guizhou province, China. Buds were gathered in March, while fully opened flowers were collected in May, coinciding with the peak blooming stage when pollen was released from the anthers for pollination.

### 2.2. Quantitative PCR

Total RNA was isolated from the bud and flowering samples. The TRIzol Reagent (Invitrogen, Carlsbad, CA, USA) was employed, adhering strictly to the manufacturer’s guidelines. The extraction process consists of multiple steps designed to guarantee the integrity and purity of the RNA. These steps include lysing the cells or tissues, separating the phases with chloroform, precipitating the RNA with isopropanol, and washing with 75% ethanol to eliminate possible contaminants like DNA and proteins. Utilizing the HiScript II Q RT SuperMix kit (Vazyme, Nanjing, China), 1 μg of total RNA underwent treatment for genomic DNA (gDNA) removal and subsequent complementary DNA (cDNA) synthesis. This was carried out in accordance with the manufacturer’s instructions. The quantitative polymerase chain reaction (qPCR) assays were conducted on the CFX ConnecTM Real-Time PCR Detection System (Bio-Rad, Hercules, CA, USA). The ChamQ Universal SYBR qPCR Master Mix (Vazyme, Nanjing, China) was used for this purpose. The EF1α gene was selected as a reference gene. The relative expression level was computed using the 2^−ΔΔCT^ method [30]. The primer sequences utilized for the study are presented in Table 1.

### 2.3. Vector Constructions

The vectors used in this study were constructed using a Lighting Cloning system (Biodragon Immunotechnology, Suzhou, China). For *35S:BSK2-FLAG* transgenic plants, the CDS of BSK2-like protein of ‘*Guichang*’ was amplified and cloned into *pCambia2306* at the *Kpn I/Sal I* vector. For Western blotting analysis, the total proteins of *35S:BSK2-FLAG* plants were extracted using lysis buffer containing 100 mM Tris-HCl, pH 7.0, 150 mM NaCl, 0.1% Nonidet P-40, 1 mM PMSF, 100 µM MG132 (obtained from MedChemExpress, Monmouth Junction, NJ, USA), and a protease inhibitor cocktail (P9599, St. Louis, MO, USA, Sigma). The supernatant was heated in 5× SDS loading buffer at 100 °C for 8 min. Subsequently, Western blotting was carried out on the proteins. Anti-FLAG antibody (1:4000 dilution, Sigma) and anti-actin antibody (1:4000 dilution, Agrisera, Vännäs, Sweden) were used for this purpose.

### 2.4. Arabidopsis Thaliana Materials and Growth Conditions

The *Arabidopsis thaliana* mutants employed in this research were of the Columbia (Col-0) genetic background. The *bsk1-2* (SALK_122120) mutants were sourced from Arashare (https://www.arashare.cn/index/Product/index.html accessed on 1 June 2024). The seeds were disinfected using 2% plant preservation mixture (Plant Cell Technology, Washington, DC, USA). They were subjected to stratification at 4 °C in the dark for two days. Subsequently, the seeds were sown on half-strength Muroshige and Skoog (MS) medium. This medium contained 1% (*w*/*v*) sucrose and 0.4% (*w*/*v*) phytagel, with the pH being adjusted to 5.7. The medium was either supplemented or not supplemented with DNA damage drugs. The plants were cultivated under long-day conditions (16 h of light and 8 h of darkness) at 22 °C in a growth chamber.

### 2.5. Alexander Staining

The rapid green staining reagent was prepared as a 0.5% solution of 95% ethanol, while the safranin staining reagent was composed of a 1% solution in 50% ethanol. The fast green–safranin dual staining technique has been widely adopted for preparing plant paraffin sections. This method stains lignified cell walls and nuclei red, whereas the more fragile cellulose-based cell walls and cytoplasm appear green. The method was modified according to a previous study [31].

### 2.6. RNA-Seq Analysis

Buds and flowers were gathered from kiwifruit plants that were 10 years old. The buds were sampled in March, while the flowers were collected in May. Three biological replicates were acquired from different individual plants. The transcriptome analyses were carried out by Novogene Co., Ltd. (Beijing, China). Briefly, the following was the workflow: The quality of the RNA sample was evaluated using the Agilent 2100 Bioanalyzer to guarantee that the RNA Integrity Number (RIN) met the strict requirements for sequencing. Indexed samples were clustered on a cBot Cluster Generation System in accordance with the TruSeq PE Cluster Kit v3-cBot-HS (Illumina, San Diego, CA, USA) protocol. After clustering, the library preparations were sequenced on the Illumina Novaseq platform, generating 150 bp paired-end reads. Subsequently, bioinformatics analysis was performed on the raw sequencing data.

### 2.7. Proteome Analysis

TMT quantitative proteomics technology was employed to conduct an analysis of the sample proteome. The proteomic samples were the same as those utilized in the transcriptomic analysis. The workflow was carried out in the following sequence: extraction of total protein; protein quality assessment; TRAQ labeling of peptides; enzymatic hydrolysis of protein and salt removal; fraction separation; LC-MS analysis; and data analysis. UHPLC-MS/MS analyses were carried out by Novogene Co., Ltd. (Beijing, China), using an EASY-nLCTM 1200 UHPLC system (Thermo Fisher, Dreieich, Germany) connected to either a Q ExactiveTM HF-X (Thermo Fisher, Germany) or an Orbitrap Exploris 480 mass spectrometer (Thermo Fisher, Germany).

## 3. Results

### 3.1. Developmental Stages of Actinidia deliciosa Cultivar ‘Guichang’

**Figure 1:**
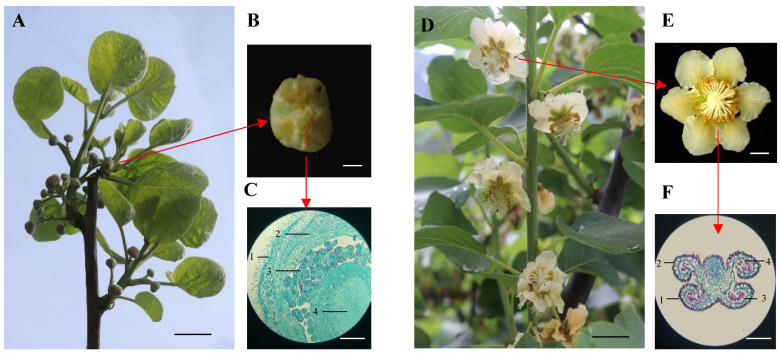
The bud and flowering stage of ‘*Guichang*’. (**A**) Small green buds merging on the branches. Scale bar, 1 cm. (**B**) Cross-sections prepared from flower buds. Scale bar, 1 cm. (**C**) Alexander staining of cross-sections from flower buds. 1, calyx; 2, petal; 3, anther; 4, stigma. Scale bar, 100 μm. (**D**) Peak flowering stage. Scale bar, 1 cm. (**E**) Cross-sections prepared from the anthers of blossoms. Scale bar, 1 cm. (**F**) Cross-sections from anthers were stained using the Alexander method. 1, epidermis; 2, fibrous layer; 3, pollen grain; 4, pollen sac. Scale bar, 100 μm.

To investigate the genetic and proteomic factors that affect the transition from the bud stage to flowering in ‘*Guichang*’, samples were obtained from both the bud and blooming phases for microscopic analysis. As shown in Figure 1A, nascent green flower buds were harvested from the branches of ‘*Guichang*’. To elucidate their internal structure, transverse sections were prepared as depicted in Figure 1B. The Alexander staining technique was employed, enabling the identification of various tissue structures such as the calyx, petals, anthers, and stigma (Figure 1C). During the peak flowering period of ‘*Guichang*’, characterized by fully expanded flowers, active pollen discharge from the anthers, and stigma readiness for pollination, samples were collected (Figure 1D). This period aligns with the optimal conditions for reproductive success in plants, consistent with findings in studies of various ornamental plant species. Similarly to the flower buds, cross-sections of pollen grains were prepared for analysis of their maturity and morphological features (Figure 1E). Additionally, the Alexander staining method was applied to these pollen sections to distinguish different cellular layers and structures (Figure 1F). These results confirmed the successful collection of ‘*Guichang*’ samples at both the bud and flowering stages.

### 3.2. Analysis of RNA-Seq Data for Transcript Splicing

After processing the raw data to filter out errors and analyze the GC content distribution, we acquired high-quality reads for further analysis (Figure S1A). A Principal Component Analysis (PCA) in a 3D plot depicted gene expression patterns during the bud stage (GF1) and bloom stage (GF2). The analysis demonstrated a clear distinction in the overall transcriptome between GF1 and GF2, with no statistically significant variation within each group (Figure S1B). To validate the experiments and sample selection, we conducted a Pearson correlation analysis, revealing R² values exceeding 0.8 for replicate samples (GRF11, GRF12, GRF13) and (GRF21, GRF22, GRF23), indicating a strong model fit and high reproducibility (Figure S1C). These findings support the reliability of the subsequent differential gene analyses.

**Figure 2:**
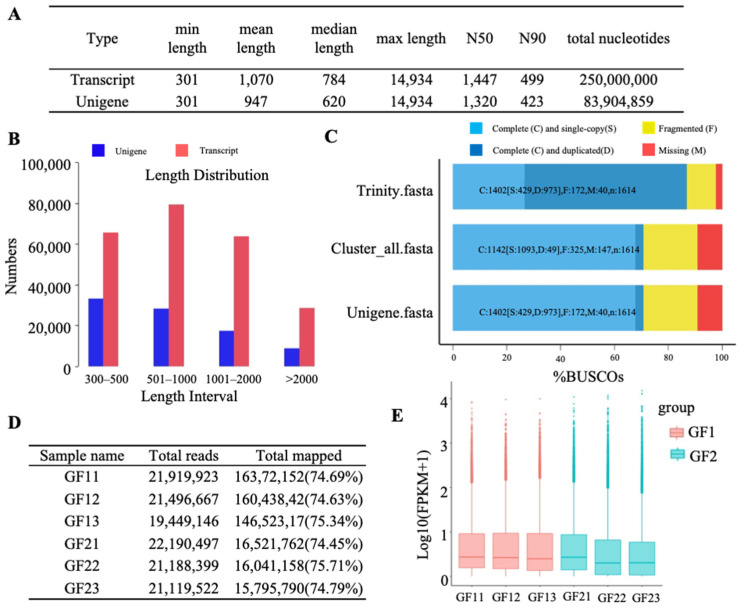
RNA-seq data analysis for transcript splicing. (**A**) Summary of spliced transcript/unigene length statistics. Min_length is the shortest spliced transcript/unigene, Mean_length is the average length, Median_length is the middle value, Max_length is the longest, N50 is the length at which half of the total transcript length is achieved in descending order, indicating assembly quality, and N90 is the length covering 90% of the total transcript length. Total_nucleotides represent the total nucleotide count in spliced transcripts/unigenes. (**B**) Distribution of unigene and transcript lengths. (**C**) BUSCO assessment results, with colors indicating different spliced transcript types, offering insights into transcriptome assembly completeness and accuracy. (**D**) The mapping ratio of clean reads to unigenes: Total Reads refers to the number of clean reads after quality control. Total Mapped represents the count of reads that are mapped to unigene sequences. The values in parentheses indicate the proportion of mapped reads relative to the total number of clean reads. (**E**) FPKM boxplot displaying the distribution of FPKM values under various experimental conditions, indicating the overall gene expression levels.

For transcriptome sequencing analysis, we assembled sequences into transcripts and performed hierarchical clustering using the Corset program, along with statistical evaluations of transcript lengths. The average length of the transcripts was 1070 bp, and the mean length of the unigenes was 947 bp (Figure 2A). The analysis of spliced transcript and unigene lengths showed that unigene lengths mainly fell within the 301–500 bp and 501–1000 bp ranges (Figure 2B). The completeness of the transcriptome assembly was evaluated using BUSCO, which identified various types of spliced transcripts, confirming their accuracy and thoroughness (Figure 2C).

For the gene expression analysis, we constructed a reference sequence from the Trinity transcriptome and aligned clean reads from each sample to this reference [32]. Reads with alignment quality scores below 10, non-unique alignments, and multi-region genome alignments were excluded. Statistical comparisons of the samples are shown in Figure 1, highlighting differences in mean values and standard deviations between the two groups. The mapping ratio of clean reads exceeded 70% across samples (Figure 2D). A boxplot of FPKM values illustrated significant disparities in expression levels between the bud and flowering stages of ‘*Guichang*’, particularly between GF1 and GF2, while variations within each group were not significant, indicating distinct gene expression patterns during these developmental stages (Figure 2E). Overall, these results confirm the reproducibility of our experimental data.

### 3.3. Phytohormones Play a Pivotal Role in the Shift from the Bud Stage to Flowering in ‘Guichang’

After acquiring the spliced transcriptome, we carried out gene functional annotation by making use of seven key databases: Nr (NCBI non-redundant protein sequences), Nt (NCBI nucleotide sequences), Pfam [33], KOG/COG (COG stands for Clusters of Orthologous Groups of proteins; KOG represents euKaryotic Ortholog Groups), Swiss-Prot (a protein sequence repository that is manually curated and reviewed), KEGG (Kyoto Encyclopedia of Genes and Genomes), and GO (Gene Ontology). Our statistical analysis revealed that the Nt database had the highest annotation success rate (Figure S2A). In our genetic analysis, we utilized five important databases, including NCBI’s NT, to identify 7105 shared genes and 13,104 unique genes (Figure S2B).

**Figure 3:**
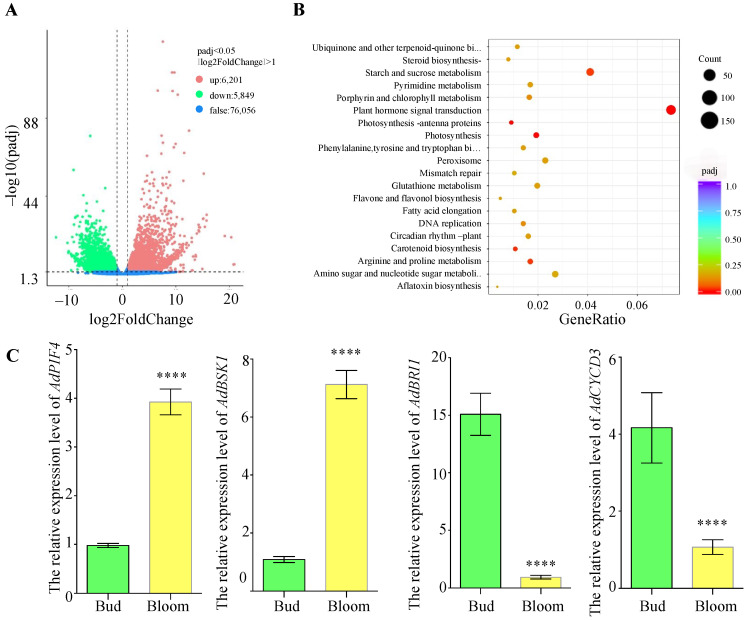
Transcriptome sequencing data analysis of the bud and flowering stages of ‘*Guichang*’. (**A**) The volcano plot shows the expression levels of differentially expressed genes. Up-regulated genes are represented by red dots, while down-regulated genes are indicated by green dots. (**B**) KEGG enrichment analysis of up-regulated genes, where the Gene Ratio represents the proportion of differentially expressed genes within a specific pathway compared to all annotated genes in that pathway. The adjusted *p*-values (Padj) range from 0 to 1, with lower values indicating greater significance in enrichment. (**C**) qPCR was used to measure the transcription levels of various genes at the bud and flowering stages, using EF1a as a reference gene. Results are expressed as mean ± SD (*n* = 3). **** *p* < 0.0001.

To analyze gene expression differences comprehensively, we utilized DESeq2 [34] to normalize the raw read counts and adjusted *p*-values for hypothesis testing, with a threshold of padj < 0.05. For each comparison group, we carried out a differential significance analysis, which resulted in a volcano plot depicting the differentially expressed genes. As shown in Figure 3A, 6201 genes were found to be up-regulated, while 5849 genes were down-regulated. Subsequently, we conducted KEGG pathway enrichment analysis on these genes, with *p*-values ranging from 0 to 1. Figure 3B indicates that the plant hormone signal transduction pathway was the most significantly enriched pathway, demonstrating regulation of Diterpenoid and brassinosteroid biosynthesis. As shown in Figure S3, *TF* and *BSK* were up-regulated, while *BRI1* and *CYCD3* were down-regulated. To verify the differential expression within the plant hormone signal transduction pathway, we cloned the CDS of these genes from ‘*Guichang*’ and performed quantitative PCR (qPCR). As shown in Figure 3C, compared to the bud stage, the relative expression levels of *AdPIF4* and *AdBSK1* in ‘*Guichang*’ increased significantly during the transition to the flowering stage. Conversely, the expression levels of *AdBAK1* and *AdCYCD3* were markedly down-regulated. These results imply that these genes might play a crucial role in the developmental transition from the bud stage to the flowering stage in ‘*Guichang*’.

### 3.4. Proteomic Analysis of ‘Guichang’ During the Bud and Flowering Stages

To investigate the protein regulatory mechanisms involved in the shift from bud to flowering stages in ‘*Guichang*’, we performed a comparative proteomic analysis of these developmental phases. By using Proteome Discoverer for database searches, we identified that Peptide Spectrum Matches (PSMs) with a confidence level of ≥99% are considered highly reliable, corresponding to a z-value of 2.576, which indicates strong statistical significance. Proteins that were identified by at least one unique peptide were also considered trustworthy, as they provided a unique identifier for protein verification. We retained only these reliable PSMs and proteins, applying FDR validation to eliminate those with an FDR greater than 1%. Our analysis identified a total of 38,584 peptides and 10,514 proteins (Table 2).

To ensure the quality of our proteomic data, we carefully assessed various parameters. The peptide length distribution, mainly between 7 and 25 residues (Figure S4A), confirmed the appropriate choice of protease. The precursor ion tolerance, a crucial measure of mass spectrometry accuracy, was nearly zero (Figure S4B), indicating excellent identification precision. The analysis of unique peptides per protein, used to evaluate the protein reliability, showed a slight upward trend (Figure S4C), reflecting significant identification of trustworthy proteins. The protein coverage, which indicates the identification accuracy, was 31.17% for the 0–0.1 range, 27.19% for 0.1–0.2, and 16.94% for the 0.2–0.3 ranges (Figure S4D), suggesting generally reliable results. The Principal Component Analysis (PCA) indicated clear proteomic differences between the bud group (GF1) and the flowering group (GF2), with minimal variation within each group (Figure S4E). The Coefficient of Variance (CV)’s Cumulative Curve showed a steep increase, indicating high reproducibility among samples (Figure S4F). Together, these findings confirm the reliability and reproducibility of our proteomic data.

**Figure 4:**
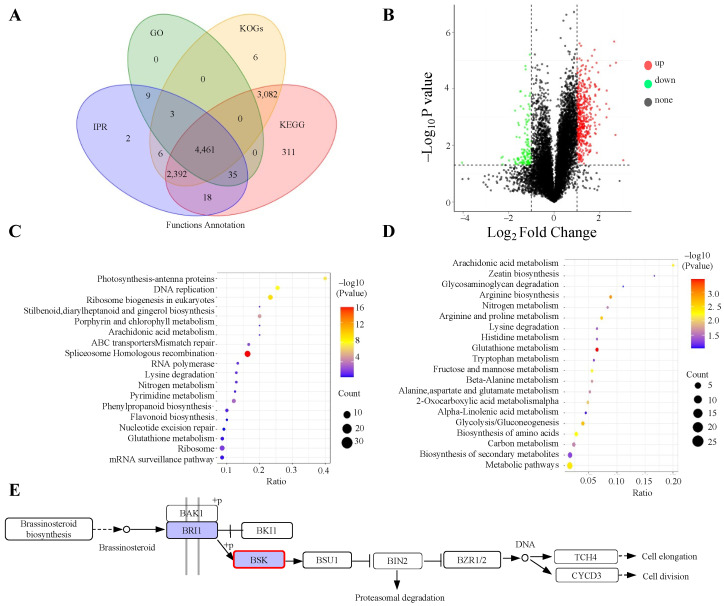
Enrichment analysis of proteomics data from the bud and flowering stages of ‘*Guichang*’. (**A**) Venn diagram showing the annotation results, created using four different databases, with colors indicating each database. (**B**) A volcano plot is presented to show the relative abundance of differential proteins. In this plot, red dots stand for up-regulated proteins, while green dots denote down-regulated proteins. (**C**) KEGG enrichment analysis of up-regulated proteins, with the ratio reflecting the number of differential proteins in a category relative to the total proteins identified in that pathway. A higher ratio signifies greater enrichment of differential proteins. The dots’ colors represent the significance of the hypergeometric test, with darker colors indicating lower *p*-values, while the dot size corresponds to the number of differential proteins in each pathway. (**D**) KEGG enrichment analysis of down-regulated proteins, where the differential ratio is calculated similarly. The dot colors indicate statistical significance, with redder colors showing smaller *p*-values. The larger the dot is, the more differential proteins are associated with that pathway. (**E**) The Brassinosteroid biosynthesis pathway shows the highest level of enrichment. Genes that are up-regulated are marked in red, while those that are down-regulated are marked in green.

Next, we annotated the functions of the identified proteins using the GO, KEGG, COG, and IPR databases. A total of 4461 proteins were found to be common across all databases, while 311 proteins were uniquely annotated in the KEGG database (Figure 4A). For the differential protein analysis, we computed the fold change (FC) by comparing the average quantitative values of biological replicates in the experimental sample with those in the reference sample. A significance threshold of *p*-value ≤ 0.05 was applied. We identified 10,488 proteins as present in both the bud and flowering stages (Table 3). A volcano plot illustrated the relative abundance of differentially expressed proteins (Figure 4B), consistent with the data in Table 3. The KEGG database helped identify key biochemical and signaling pathways related to the differentially expressed proteins. As shown in Figure 4C, proteins that were involved in spliceosome Homologous recombination were significantly up-regulated, whereas those that were involved in glutathione metabolism were significantly down-regulated (Figure 4D). Even though the plant hormone signal transduction pathway did not exhibit the highest enrichment, we discovered that BSK was up-regulated (Figure 4E). Overall, these results indicate that the BR pathway is essential for the transition from the bud stage to the flowering stage in ‘*Guichang*’.

### 3.5. Correlation Analysis of Protein and Transcriptome Derived from the Bud and Flowering Phases of ‘Guichang’

**Figure 5:**
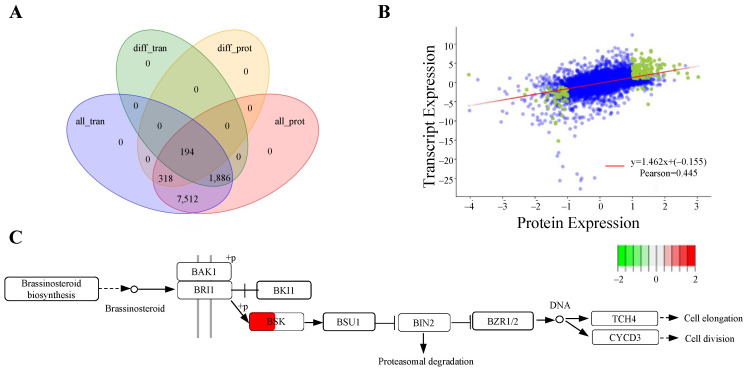
Analysis of expression regulation in transcriptome and proteome derived from bud and flowering stages of ‘*Guichang*’. (**A**) A Venn diagram showing the overlap and distinct features of expression regulation in transcriptome and proteome. “All_tran” refers to all identified genes in the transcriptome, “diff_tran” refers to differentially expressed genes, “all_prot” indicates all identified proteins in the proteome, and “diff_prot” signifies differentially expressed proteins. This comparison helps identify common and unique genes or proteins in each category. (**B**) Correlation analysis between transcriptome and proteome expression levels. Each dot represents a protein, with green dots indicating proteins showing significant differential expression and blue dots representing proteins without significant differences. (**C**) The KEGG enrichment analysis was conducted on metabolic pathways by integrating transcriptomic and proteomic data. Genes or proteins are represented by boxes, where each box is divided. The left part of the box presents the log2 fold change (log2FC) of the protein, and the right part shows the log2FC of the transcript. Up-regulation is indicated by red boxes, while down-regulation is indicated by green boxes.

To achieve a comprehensive overview of the expression profiles, we performed a detailed analysis of the gene and protein expression levels. By integrating mRNA data from transcriptomics with protein data from proteomics, we aimed to elucidate their intricate relationships and regulatory mechanisms. A Venn diagram constructed during this analysis revealed that 194 genes were shared among the four groups (Figure 5A). We also carried out a correlation analysis of the fold changes in genes and proteins detected by both transcriptomics and proteomics, yielding a Pearson correlation coefficient of 0.445, which indicates a moderate positive relationship between transcription and protein expression levels (Figure 5B). Furthermore, KEGG pathway analysis indicated a positive correlation for BSK between gene and protein expressions (Figure 5C). These findings suggest that AdBSK may play a crucial role in the transition from the bud stage to the flowering stage in ‘*Guichang*’.

### 3.6. BSK of ‘Guichang’ (AdBSK) Promotes Flowering in Arabidopsis

To study the role of AdBSK in ‘*Guichang*’, we cloned the *AdBSK1* gene for ‘*Guichang*’ based on unigene and CDS prediction data. We then created the 35S:AdBSK1-FLAG plasmid and introduced it into the *bsk1-2* mutant of *Arabidopsis*, resulting in the transgenic plants *35S:AdBSK1-FLAG/bsk1-2*.

**Figure 6:**
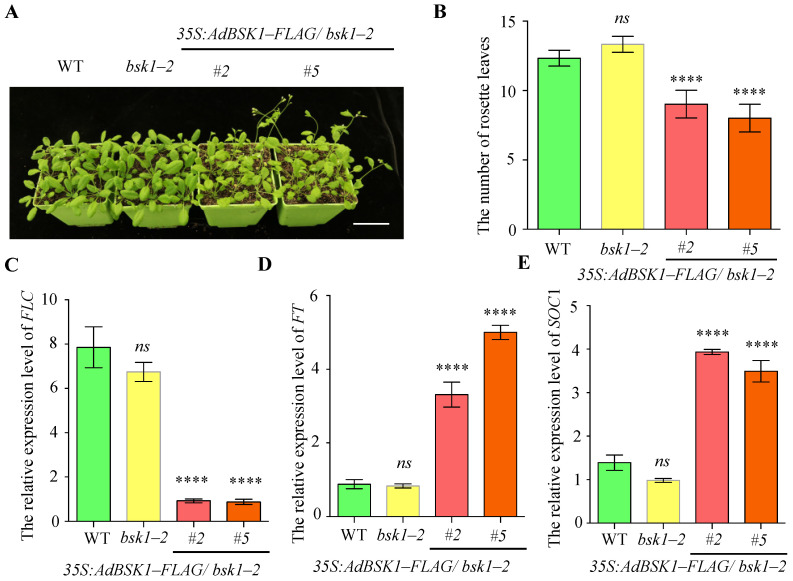
AdBSK1 promotes plants flowering earlier in *Arabidopsis.* (**A**) Phenotypic analysis of flowering in the WT, *bsk1-2*, and *35S:AdBSK1-FLAG/bsk1-2* plants. Four-week-old seedlings were grown on soil under natural conditions. Plant images (**A**) are shown. Scale bar, 1 cm. (**B**) Rosette leaf number in the WT, *bsk1-2*, and *35S:AdBSK1-FLAG/bsk1-2* plants. One-way ANOVA was employed to determine the statistical significance between the WT and other genotype (*n* = 10). (**C**) The relative expression levels of *AdFLC* during the flowering stage in the WT, *bsk1-2*, and *35S:AdBSK1 -FLAG/ bsk1-2*. The relative expression level of a gene was determined by qPCR with EF1α as a reference gene. The data are presented as means ± standard deviation (SD) (*n* = 3). (**D**) Similarly, for *AdFT*, its relative expression levels during the flowering stage in WT, *bsk1-2*, and *35S:AdBSK1-FLAG/bsk1-2* were examined. The relative expression level of this gene was also determined by qPCR using EF1α as a reference gene. The data are shown as means ± SD (*n* = 3). (**E**) Regarding *AdSOC1*, its relative expression levels during the flowering stage in the WT, *bsk1-2*, and *35S:AdBSK1-FLAG/bsk1-2* were analyzed. The relative expression level of the gene was determined by qPCR with EF1α as a reference gene. The data are expressed as means ± SD (*n* = 3). **** *p*< 0.0001, *ns* = not significant.

The *bsk1-2* mutant was confirmed by PCR analysis in Figure S5A, while the protein level of BSK1 in *35S:AdBSK1-FLAG* plants was verified by Western blotting analysis (Figure S5B). Subsequently, we performed a genotype analysis on the WT, *bsk1-2* mutant, and *35S:AdBSK1-FLAG/ bsk1-2* plants to assess the role of *AdBSK1* in plant development. As shown in Figure 6A, compared with both the WT and the *bsk1-2* mutant, the *35S:AdBSK1-FLAG/bsk1-2* plants showed an earlier flowering phenotype. Conversely, due to the functional redundancy provided by other AdBSK homologs, no significant differences in flowering time were detected between the WT and the *bsk1-2* mutant. After counting the number of rosette leaves, it was found that the number of rosette leaves in the *bsk1-2* mutant was similar to that of the WT. However, the *35S:AdBSK1-FLAG/bsk1-2* plants had a significantly lower number of rosette leaves (Figure 6B). To investigate the mechanism underlying AdBSK1-mediated promotion of flowering, we cloned the CDS regions of *FLC*, *FT*, and *SOC1* and performed qPCR analysis. As shown in Figure 6C, the transcription levels of *FT* and *SOC1* were significantly increased, while the *FLC* levels were notably decreased in the *35S:AdBSK1-FLAG/bsk1-2* plants. These findings suggest that AdBSK1 of ‘*Guichang*’ promotes earlier flowering by regulating the expressions of *FLC*, *FT*, and *SOC1*.

## 4. Discussion

In this study, we carried out investigations to reveal the molecular mechanisms that underlie flowering in ‘*Guichang*’ using transcriptomic and proteomic analyses. Our results indicated that the transition from bud development to flowering is governed by plant hormone signaling pathways. At the transcriptional level, we observed up-regulation of *AdBSK* and *AdPIF4*, while *AdBRI1* and *AdCYCD3* were down-regulated to facilitate flowering. At the protein level, only AdBSK was regulated. Additionally, over-expressing AdBSK from ‘*Guichang*’ in *Arabidopsis* resulted in decreased *FLC* expression and increased levels of *FT* and *SOC1*, thereby accelerating the earlier flowering in the plants.

Our findings indicate that the signaling pathways of plant hormones, particularly gibberellins, play a vital role in the transformation from bud to flower. This discovery is consistent with previous studies that emphasized the important part of hormone signaling in plant growth and development [35,36,37]. The shift from the vegetative growth phase to the reproductive stage in plants is a complicated process that is regulated by numerous internal and external factors. Hormones such as auxin are indispensable for the development of floral organs [38,39]. Notably, we found increased expression levels of the transcription factor *AdPIF4* (Figure 3C and Figure S4A), which is consistent with its role in promoting flowering [40,41]. In contrast, the transcript levels of *AdCYCD3* decreased during the flowering stage, suggesting its role in inhibiting flowering (Figure 3C and Figure S4B). Nevertheless, no notable alterations were found in the protein levels of AdCYCD3. This implies that in ‘*Guichang*’, multi-level regulation takes place at the post-transcriptional and post-translational levels.

The moderate correlation (Pearson = 0.445) also supports the discordance between the gene and protein expression levels (Figure 5C), and several biologically significant factors likely contribute to this discrepancy. Firstly, variations in mRNA stability, translation initiation efficiency, and ribosomal pausing affect mRNA translation rates, regardless of transcript abundance [42,43]. Secondly, post-translational modifications (PTMs) like phosphorylation, ubiquitination, and glycosylation impact proteins’ activity, stability, and turnover, meaning that detected protein levels may not solely reflect the synthesis rates [44]. Thirdly, proteins have varying half-lives; some are quickly degraded (e.g., through the ubiquitin–proteasome system), while others are stable, resulting in accumulation or depletion that is not directly linked to transcription levels [45]. Additionally, differences in sensitivity, dynamic range, and normalization methods between transcriptomic and proteomic techniques can lead to perceived inconsistencies [46]. Future studies that combine multi-omics approaches and assess protein turnover rates could provide deeper insights into the underlying regulatory mechanisms.

In the BR pathway, both transcriptome and proteome analyses showed up-regulation of BSK, indicating that BSK enhances flowering at both the transcript and protein levels. To confirm the role of AdBSK in ‘*Guichang*’, we over-expressed the AdBSK1 of ‘*Guichang*’ in *Arabidopsis* (Figure S5B) and performed genotype analysis. The results demonstrated that the over-expression of AdBSK leads to earlier flowering (Figure 6A,B), suggesting that AdBSK1 positively regulates flowering in ‘*Guichang*’. In the *35S:AdBSK1-FLAG/bsk1-2* plants, *FT* and *SOC1* were significantly up-regulated, while *FLC* was notably down-regulated (Figure 6C–E), suggesting that AdBSK1 promotes the transcription of *FT* and *SOC1* while repressing that of *FLC*. Our findings suggest that AdBSK1 plays a role in regulating flowering through *FT*, *SOC1*, and *FLC*.

BSK1 modulates BR-mediated growth and immune responses, which have been well studied in *Arabidopsis* [47,48,49]. In maize (*Zea mays*), ZmBSK1 [50] and BSK1 from Kentucky bluegrass (*Poa pratensis*) [51] enhance drought resistance, while GmBSK1 in soybean (*Glycine max*) [52] and SoBSKs in Spinach (*Spinacia oleracea*) [53] improve the plants’ response to temperature stress. However, studies on BSK1 in other fruit crops remain limited. A recent study found that MdBSK1 in apple enhances the resistance to *Botryosphaeria dothidea* [54]. In this study, AdBSK1 from ‘*Guichang*’ kiwifruit promoted flowering in *Arabidopsis* by inhibiting *FLC* and activating *FT*/*SOC1*, highlighting a new role for BSKs in the floral transition. This discovery offers insights into the functional diversity of BSK1, suggesting that AdBSK1 may act as a developmental regulator in kiwifruit, balancing growth and defense by accelerating reproduction under optimal conditions.

In summary, our study provides a comprehensive understanding of the molecular mechanisms underlying flowering in *Actinidia deliciosa*, expanding on recent advancements in plant flowering regulation. Although we discovered an intricate regulatory network that encompasses plant hormone signaling, transcription factors, and various proteins, additional research is essential to delve into the specific molecular mechanisms. Moreover, it is necessary to explore the potential applications of our research results for improving kiwifruit production.

## 5. Conclusions

The complex molecular mechanisms regulating the shift from bud to flowering in ‘*Guichang*’ were clarified through a combination of transcriptomic and proteomic analyses. Our findings highlight the essential roles of BR and GA signaling pathways in this vital developmental process. We identified key genes such as *AdBSK1*, *AdPIF4*, *AdBRI1*, and *AdCYCD3* as significant regulators, with *AdBSK1* being recognized as a major regulator at both the transcriptional and proteomic levels. Functional tests conducted on Arabidopsis showed that the over-expression of AdBSK1 leads to earlier flowering. This is achieved by down-regulating *FLC* and up-regulating *FT* and *SOC1*, thus verifying its conserved function in the regulation of flowering.

Our multi-omics analysis showed a moderately positive correlation (Pearson coefficient: 0.445) between the expression levels of transcripts and proteins. This finding emphasizes the complex characteristics of post-transcriptional regulation during the flowering process. Changes in transcription for *AdPIF4*, *AdBRI1*, and *AdCYCD3* pointed to their involvement in promoting and inhibiting flowering, while proteomic data emphasized the critical role of BR signaling, particularly through AdBSK1. These findings enhance our understanding of flowering processes in perennial fruit crops and provide practical insights for manipulating flowering time to improve the yield and fruit quality.

This study addresses a significant knowledge gap in horticultural science by connecting molecular pathways to agronomic traits in ‘*Guichang*’. For future research endeavors, the emphasis should be placed on verifying the regulatory functions of candidate genes in kiwifruit. Additionally, it is essential to delve into the interactions between hormone pathways and apply these research results to actual field conditions. Our study highlights the potential of omics-based approaches to optimize flowering time in economically important crops, offering a framework for enhancing cultivation techniques and breeding strategies.

## Figures and Tables

**Table 1:** List of primers.

Name	Sequence (5′-3′)	Function
*35S:AdBSK1-FLAG*	F: ATGGGTTGTTGTCAATCCTTG	cloning
*35S:AdBSK1-FLAG*	R: AGATCCTCTGCCGCCT	cloning
LP	TAGGCCATGTGCCATTTACTC	*bsk1-2*
RP	TTGCAAGTCAACTCCATACCC	*bsk1-2*
Lbe	GCGTGGACCGCTTGCTGCAACT	*bsk1-2*
*Ad*PIF4	F: GAACACAGACGCAGCC	qPCR
*Ad*PIF4	R: CTCACCAACCTAGTGGTCC	qPCR
*Ad*BSK1	F: GAACACAGACGCAGCC	qPCR
*Ad*BSK1	R: TGTGTCTCAAGAATCAAGATCCT	qPCR
*Ad*BRI1	F: AAGCCGGGTCAGGGATA	qPCR
*Ad*BRI1	R: GCTCTGTTTCTAACTCTCATAATTTTC	qPCR
*Ad*CYCD3	F: TGCAACCCACCAACGTC	qPCR
*Ad*CYCD3	R: GCTTTCGATTATGGAGTGGCTA	qPCR
*Ad*FLC	F: TGAAAGAAGAGAACCAGGTTTTG	qPCR
*Ad*FLC	R: CGATTTAAGGTGGCTAATTAAGTAGT	qPCR
*Ad*FT	F: GCGAGTTTGCTGAGATCTACA	qPCR
*Ad*FT	R: GCCATCTAAAGTCTTCTTCCTCC	qPCR
*Ad*SOC1	F: AAACGAGAAGCTCTCTGAAAAGT	qPCR
*Ad*SOC1	R: ACTTTTCAGAGAGCTTCTCGTTT	qPCR

**Table 2:** Overview of protein identification.

Total Spectra	Matched Spectrum	Peptide	Identified Protein	ALL
358,695	81,966	38,584	10,514	10,488

Generally, the numbers of peptides and proteins were identified. The term “Total spectra” denotes the overall quantity of secondary spectra. “Matched spectrum” refers to the number of spectra that have been successfully matched to a peptide sequence. “Peptide” indicates the count of identified peptides, and “Identified protein” represents the number of proteins that have been recognized. “ALL” stands for the total number of proteins that can be quantified across all samples.

**Table 3:** Overview of differential protein analysis.

Compared Samples	Num. of Total Quant	Regulated Type	Fold Change > 1.2	Fold Change > 1.3	Fold Change > 1.5	Fold Change > 2.0
GF1. vs. GF2	10,488	up-regulated	2652	2204	1391	413
		down-regulated	1014	811	462	117

The differential protein analysis results involve a comparison between the bud stage samples and the flowering stage samples of ‘*Guichang*’. The total number of quantified proteins encompasses those that were detected in both sample groups. Proteins that are specific to one group do not signify up-regulation or down-regulation, since they are only present in either the bud stage or the flowering stage.

## Data Availability

The data presented in this study are openly available in NCBI BioProject at https://www.ncbi.nlm.nih.gov/bioproject/1271994, accessed on 1 April 2025; the reference number is PRJNA1271994.

## Supplementary Materials

The following supporting information can be downloaded at: https://www.mdpi.com/article/10.3390/genes16070760/s1, Figure S1: Quality control of transcriptome sequencing data.; Figure S2: The functional annotation of genes; Figure S3: The Enrichment analysis of transcriptome sequencing data derived from bud and flowering stage of ‘*Guichang*’; Figure S4: Quality control of TMT quantitative proteomics data; Figure S5: Identification of genotype in plants.

## Abbreviations

The following abbreviations are used in this manuscript:

‘*Guichang*’	*Actinidia deliciosa cultivar* ‘*Guichang*’
GA	Gibberellin
ABA	Abscisic Acid
BRs	Brassinosteroids
BSK1/2	Brassinosteroid-Signaling Kinase 1/2
BRI1	Brassinosteroid Insensitive 1
BIN2	Brassinosteroid-Insensitive 2
BAK1	BRI1-Associated Receptor Kinase 1
BZR1	Brassinazole-Resistant 1
BES1	BRI1-EMS-Suppressor 1
SOC1	Suppressor of Overexpression of CO 1
PIF3/4	Phytochrome Interacting Factor 3/4
UVR8	UV RESISTANCE LOCUS 8
CRY1/2	CRYPTOCHROME 1/2
FT	Flowering Locus T
CO	CONSTANS
qPCR	Quantitative PCR
GF1	The budding stage of ‘*Guichang*’
GF2	The flowering stage of ‘*Hanhong*’
