# Supplementary material for: AdBSK1-Mediated Hormone Signaling Regulates Flowering Transition in Actinidia deliciosa ‘Guichang’
Source: Genes (Basel). 2025 Jun 28;16(7):760. doi: 10.3390/genes16070760 (PMC12294449; doi:10.3390/genes16070760)
Supplement: Supplementary file 1 [file genes-16-00760-s001.zip › genes-3669314-supplementary.pdf]

Supplemental Figures

A

sample	library	raw_ reads	raw_ bases	clean_ reads	clean_ bases	error_ rate	Q20	Q30	GC_ pct
GF11	GF11	22447448	6.73G	21919923	6.58G	0.03	97.66	93.61	46.53
GF12	GF12	21927291	6.58G	21496667	6.45G	0.03	97.51	93.32	46.52
GF13	GF13	19966398	5.99G	19449146	5.83G	0.03	97.69	93.74	46.75
GF21	GF21	22512735	6.75G	22190497	6.66G	0.03	97.63	93.55	46.55
GF22	GF22	21577828	6.47G	21188399	6.36G	0.03	97.55	93.37	46.65
GF23	GF23	21661871	6.5G	21119522	6.34G	0.03	97.61	93.55	46.19

B

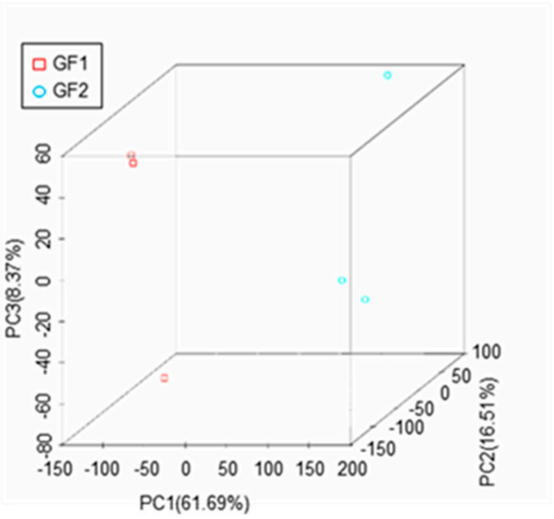

C

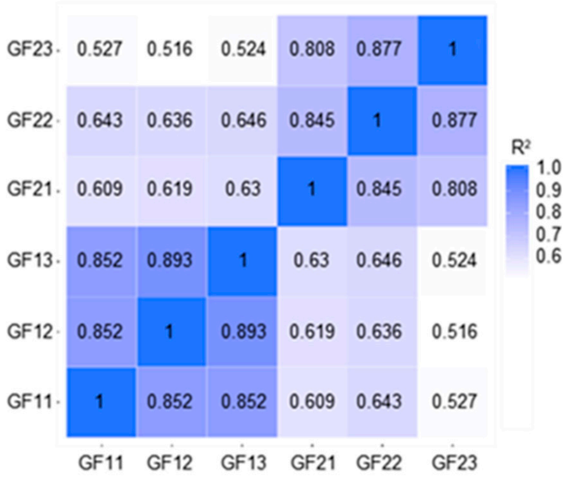

Figure S1. Quality control of transcriptome sequencing data. (A) Summary of sequencing data quality of samples. GF11, GF12, GF13 represent three independent experiment of RNA-seq of bud. GF21, GF22, GF23 represent three independent experiment of RNA-seq of bloom. Sample is sample name. Library is library number. Raw_reads is the number of reads in the raw data. Raw_bases is the number of bases in the raw data (raw base = raw reads * 150bp). Clean_reads is the number of reads after filtering the raw data. Clean_bases is the number of bases after filtering the raw data (clean base = clean reads * 150bp). Error_rate is the overall sequencing error rate of the data. Q20 is the percentage of bases with a Phred value greater than 20 among the total bases. Q30 is the percentage of bases with a Phred value greater than 30 among the total bases. GC_pct is the percentage of G and C among the four bases in clean reads. (B) PCA 3D Plot showing the gene expression patterns at the flower buds (GF1) and flower buds (GF2). PC1 and PC2 represent the first principal component and the second principal component, respectively. (C) Pearson correlation between samples. The abscissa is log10(FPKM + 1) of different sample, the ordinate is log10(FPKM + 1) of different sample, R2 represent the square of the Pearson correlation coefficient.

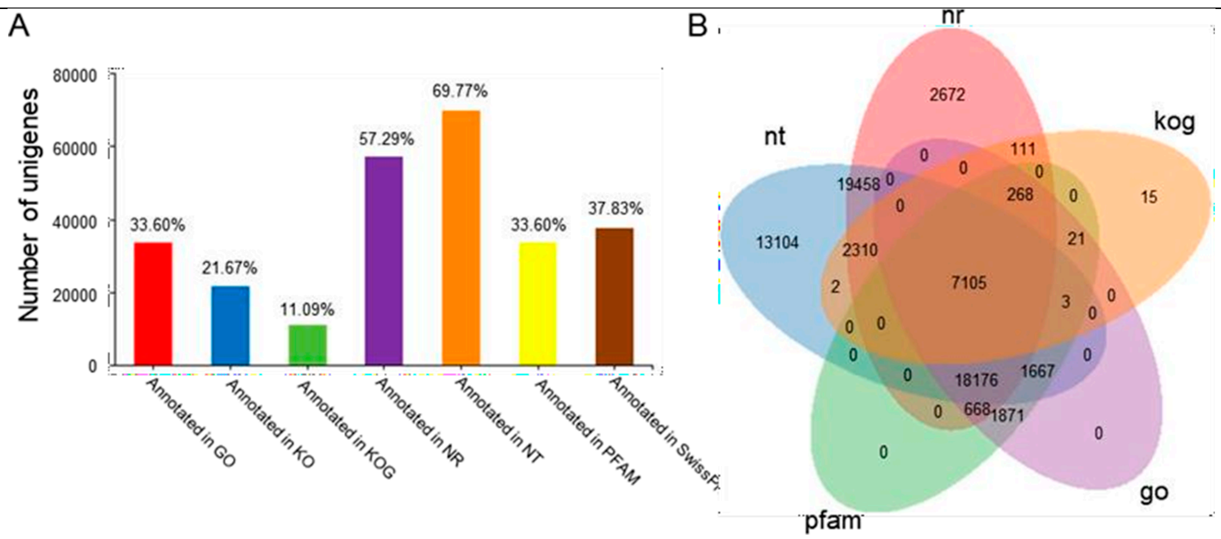

Figure S2. The functional annotation of genes. (A) Annotation of seven databases. Seven databases includes Nr, Nt, Pfam, KOG/COG, Swiss-prot, KEGG, and GO. Number of Unigenes is the number of Unigenes annotated to the database Gene. Percentage is the proportion of Genes annotated to the database among all Genes. (B) Venn diagram of annotation results .The Venn diagram was drawn based on five databases. Different colors represent different databases.

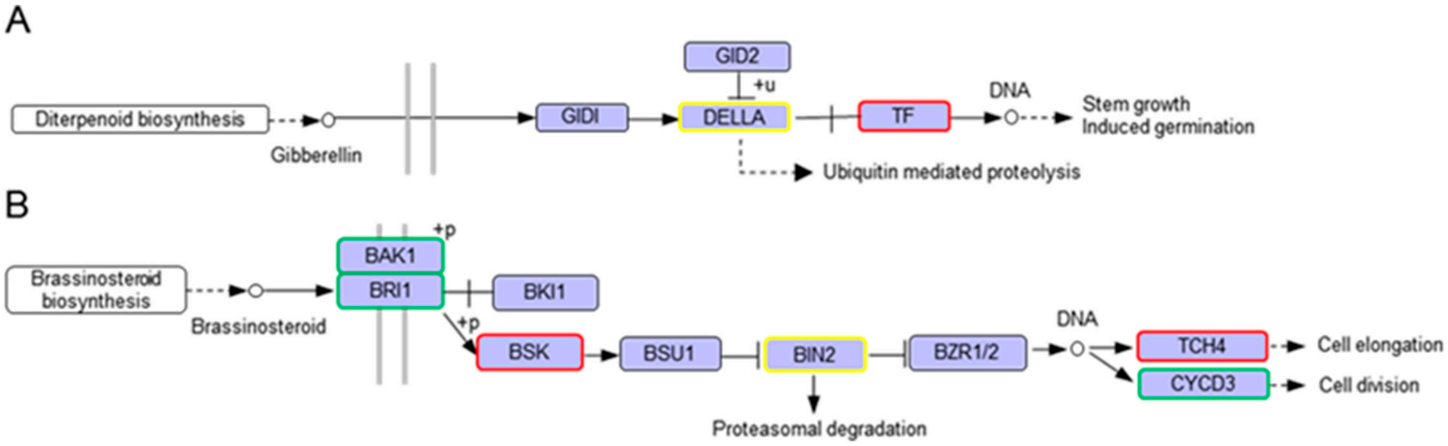

Figure S3. The Enrichment analysis of transcriptome sequencing data derived from bud and flowering stage of A. Guichang. (A) The most enriched pathway of Diterpenoid biosynthesis pathway. Up-regulated genes are marked in red. Down-regulated genes are marked in green. Both up- and down-regulated genes are marked in yellow. (B) The most enriched pathway of Tryptophan metabolism pathway. Up-regulated genes are marked in red. Down-regulated genes are marked in green. Both up- and down-regulated genes are marked in yellow.

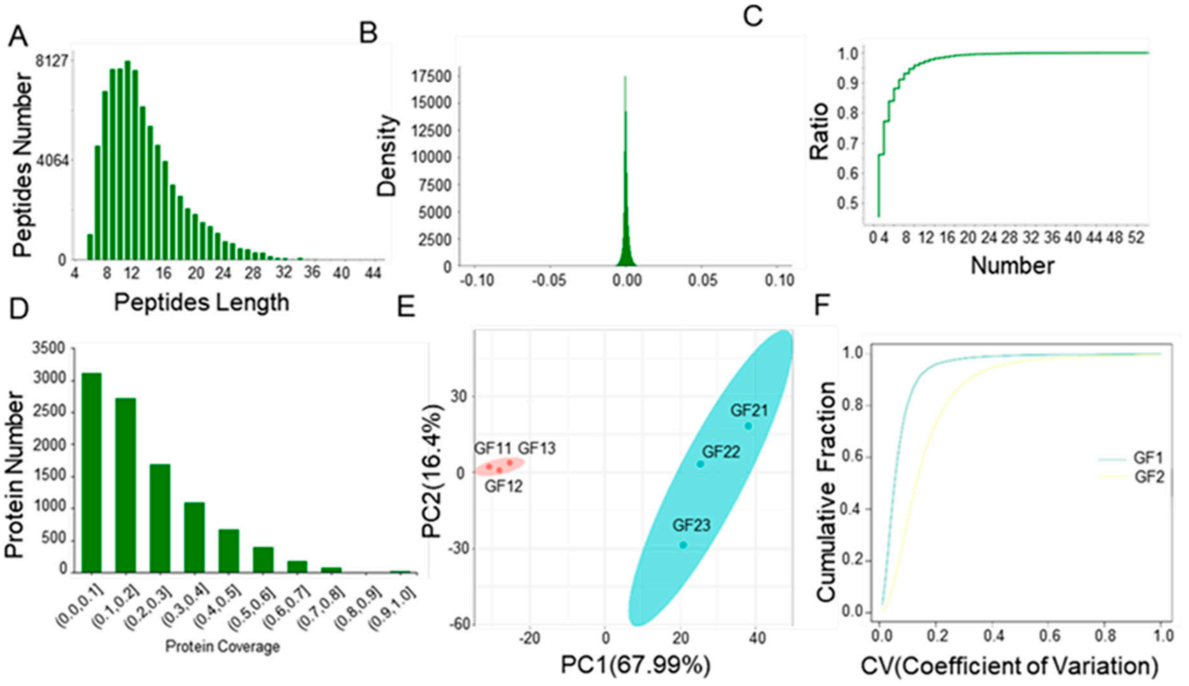

Figure S4. Quality control of TMT quantitative proteomics data. (A) The distribution of Peptide length. For conventional complex samples, the peptide length is mainly distributed between 7-25. (B) Precursor lon Tolerance. The more concentrated the peak shape is around 0, the smaller the mass deviation is. (C) The distribution of Unique peptide numbers. The curve increases more slowly, the number of Unique peptides is larger and more reliable proteins are identified. (D) The distribution of protein coverage. The identification coverage of proteins can indirectly reflect the overall accuracy of the identification results. (E)) PCA Plot shows the overall protein differences among the group of flower buds (GF1) and flower buds (GF2) . PC1 and PC2 represent the first principal component and the second principal component, respectively. (F) CV (Coefficient of Variance) Cumulative Curve shows the cumulative plot of the CV values of all proteins in the corresponding samples. The faster the curve rises, the better the overall repeatability of the samples.

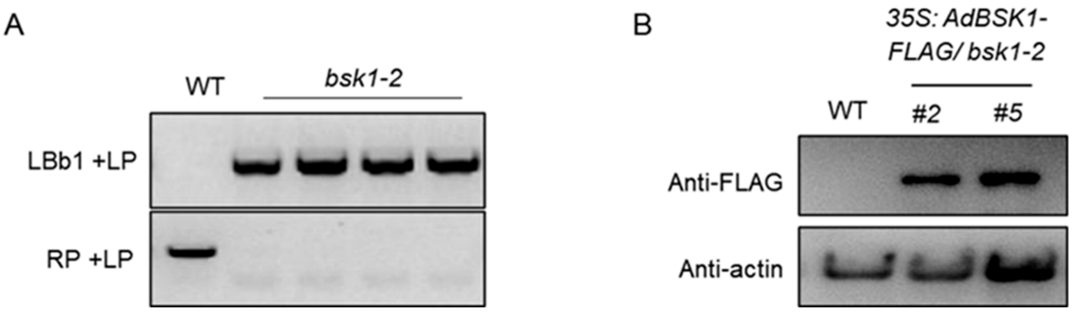

Figure S5. Identification of genotype in plants. (A) Identification of T-DNA insertion in the bsk1-2 mutant. PCR identification of mutant genotypes using leaf DNA templates. (B) The protein level of AdBSK1 in 35S:AdBSK1-FLAG/ bsk1-2 was confirmed by western blotting.
